# Ets1 and IL17RA cooperate to regulate autoimmune responses and skin immunity to *Staphylococcus aureus*

**DOI:** 10.3389/fimmu.2023.1208200

**Published:** 2023-08-23

**Authors:** Michael Battaglia, Alex C. Sunshine, Wei Luo, Richard Jin, Alifa Stith, Matt Lindemann, Lloyd S. Miller, Satrajit Sinha, Elizabeth Wohlfert, Lee Ann Garrett-Sinha

**Affiliations:** ^1^ Department of Biochemistry, State University of New York at Buffalo, Buffalo, NY, United States; ^2^ Department of Microbiology and Immunology, State University of New York at Buffalo, Buffalo, NY, United States; ^3^ AESKU Diagnostics, Buffalo, NY, United States; ^4^ Department of Dermatology, Johns Hopkins University School of Medicine, Baltimore, MD, United States

**Keywords:** autoimmunity, immunodeficiency, *Staphylococcus aureus*, autoantibodies, ETS1, IL17RA, dendritic epidermal T cell

## Abstract

**Introduction:**

Ets1 is a lymphoid-enriched transcription factor that regulates B- and Tcell functions in development and disease. Mice that lack Ets1 (Ets1 KO) develop spontaneous autoimmune disease with high levels of autoantibodies. Naïve CD4 + T cells isolated from Ets1 KO mice differentiate more readily to Th17 cells that secrete IL-17, a cytokine implicated in autoimmune disease pathogenesis. To determine if increased IL-17 production contributes to the development of autoimmunity in Ets1 KO mice, we crossed Ets1 KO mice to mice lacking the IL-17 receptor A subunit (IL17RA KO) to generate double knockout (DKO) mice.

**Methods:**

In this study, the status of the immune system of DKO and control mice was assessed utilizing ELISA, ELISpot, immunofluorescent microscopy, and flow cytometric analysis of the spleen, lymph node, skin. The transcriptome of ventral neck skin was analyzed through RNA sequencing. S. aureus clearance kinetics in in exogenously infected mice was conducted using bioluminescent S. aureus and tracked using an IVIS imaging experimental scheme.

**Results:**

We found that the absence of IL17RA signaling did not prevent or ameliorate the autoimmune phenotype of Ets1 KO mice but rather that DKO animals exhibited worse symptoms with striking increases in activated B cells and secreted autoantibodies. This was correlated with a prominent increase in the numbers of T follicular helper (Tfh) cells. In addition to the autoimmune phenotype, DKO mice also showed signs of immunodeficiency and developed spontaneous skin lesions colonized by Staphylococcus xylosus. When DKO mice were experimentally infected with Staphylococcus aureus, they were unable to clear the bacteria, suggesting a general immunodeficiency to staphylococcal species. γδ T cells are important for the control of skin staphylococcal infections. We found that mice lacking Ets1 have a complete deficiency of the γδ T-cell subset dendritic epidermal T cells (DETCs), which are involved in skin woundhealing responses, but normal numbers of other skin γδ T cells. To determine if loss of DETC combined with impaired IL-17 signaling might promote susceptibility to staph infection, we depleted DETC from IL17RA KO mice and found that the combined loss of DETC and impaired IL-17 signaling leads to an impaired clearance of the infection.

**Conclusions:**

Our studies suggest that loss of IL-17 signaling can result in enhanced autoimmunity in Ets1 deficient autoimmune-prone mice. In addition, defects in wound healing, such as that caused by loss of DETC, can cooperate with impaired IL-17 responses to lead to increased susceptibility to skin staph infections.

## Introduction

The Ets1 transcription factor is highly expressed in B and T cells and regulates their differentiation (1). Mice lacking Ets1 (Ets1 KO mice) develop autoimmune disease, with similarities to human lupus (2). Indeed, genome-wide association studies (GWASs) have associated single-nucleotide polymorphisms (SNPs) in the human *ETS1* gene locus with an increased susceptibility to systemic lupus erythematosus (SLE) (3–5). The levels of Ets1 mRNA are reduced in peripheral blood mononuclear cells (PBMCs) of lupus patients and are inversely correlated with the serum titers of autoantibodies against double-stranded DNA (dsDNA) (4, 6, 7). SNPs in the *ETS1* locus are also associated with susceptibility to rheumatoid arthritis, psoriasis, and ankylosing spondylitis, as well as multiple other autoimmune and inflammatory diseases (8–13).

The autoimmune phenotype of Ets1 KO mice has been attributed to changes in both the B- and T-cell compartments. Indeed, Ets1 KO mice have increased percentages of activated B cells, class-switched B cells, and antibody-secreting plasma cells (2, 14, 15). This is coupled with increased levels of serum IgM, IgG1, and IgE (16, 17). Serum of Ets1 KO mice also has high titers of autoantibodies to dsDNA and other autoantigens (2, 17). *In vitro*, Ets1 KO B cells have been shown to differentiate more readily into plasma cells when exposed to Toll-like receptor stimulation (2, 18, 19). The increase in plasma cells *in vivo* and the production of autoantibodies have a B cell-intrinsic component, as shown by the generation of mixed bone marrow chimeras and B cell-specific knockout of Ets1 (18, 20).

In the T-cell compartment, Ets1 KO mice have a number of important alterations, including an increase in the number of T cells with a memory phenotype (17, 21), increased differentiation into T follicular helper type 2 (Tfh2) cells that secrete high levels of IL-4 (22), and enhanced generation of Th17 cells (23). Furthermore, SLE patients carrying Ets1 risk alleles tend to have higher serum IL-17 levels than patients lacking these risk alleles (24). Ets1 KO mice have also been reported to have fewer Foxp3^+^ CD25^+^ regulatory T (Treg) cells in the spleen and thymus and the Treg cells that do develop have impaired suppressive activity (17). This is likely due to a role for Ets1 in binding to regulatory elements in the *Foxp3* gene to promote transcription (17, 25). Together, these alterations in T-cell differentiation contribute to the progression of autoimmune disease in Ets1 KO mice, as evidenced by knockout of Ets1 specifically in T cells, which results in autoimmune disease (22).

Th17 cells and IL-17 have both been shown to be increased in multiple autoimmune diseases and to play roles in driving inflammatory pathogenesis. In SLE, increased levels of IL-17 have been positively correlated with disease severity and negatively correlated with response to immunosuppressive treatment (26). In addition to its described role in autoimmune diseases, IL-17 is also important in immune responses against pathogens such as *S. aureus* (27) and *Candida albicans* (28). The protective role of IL-17 against certain pathogens is ascribed to its ability to induce the production of antimicrobial peptides that kill bacteria and chemokines involved in recruiting neutrophils (29–31). To test the role of IL-17 in promoting autoimmune disease in Ets1 KO mice, we crossed Ets1 KO mice to IL-17 receptor A subunit (IL17RA) KO mice to generate double knockout (DKO) mice. The resulting mice can produce IL-17 but are unable to respond to it. Given the aforementioned role of IL-17, we anticipated that autoimmune disease in these mice might be less severe than that in Ets1 KO mice. However, to our surprise, DKO mice displayed worse autoimmune disease than control mice, and this was coupled with increased numbers of Tfh cells, germinal center B cells, class-switched B cells, memory B cells, and plasma cells. Furthermore, they demonstrated a high susceptibility to staphylococcal infections of the skin. γδ T cells are known to be crucial for anti-staphylococcal skin immune responses. We found that mice lacking Ets1 lack the dendritic epidermal T cell (DETC) subset of γδ T cells. To probe the mechanistic role of DETC in staphylococcal infection, we depleted DETCs from IL17RA KO mice, which led to enhanced susceptibility to skin staphylococcal infections. Thus, DETCs, whose development is dependent on Ets1, cooperate with IL-17 signaling to regulate skin immune responses to staphylococcal infection. The persistent skin colonization with high levels of staph bacteria likely induces increased immune cell activation and may contribute to the development of increased autoimmune responses in DKO mice as compared to Ets1 KO mice.

## Methods

### Mice

The following mouse strains were used in this report: Ets1^-/-^ (Ets1 KO) (2), IL17RA^-/-^ (IL17RA KO) (32), and Ets1^-/-^IL17RA^-/-^ (DKO mice) as well as wild-type (WT) littermate controls that were obtained by breeding heterozygotes of the above strains. IL17RA KO mice used in this study were obtained from Amgen, Inc (31).. Since the loss of Ets1 results in perinatal lethality on an inbred C57BL/6 background (33), all mice were maintained on a mixed C57BL/6 × 129Sv genetic background. Blood was obtained under anesthesia using either retro-orbital bleed or cardiac puncture. Animals used for experiments were euthanized with CO_2_ induction followed by cervical dislocation. Mice were housed under specific pathogen-free (SPF) conditions for the duration of the studies. All studies were approved by the University at Buffalo Institutional Animal Care and Use Committee (IACUC).

### Flow cytometry

Spleen and lymph nodes were isolated from WT, Ets1 KO, IL17RA KO, and DKO mice, and single-cell suspensions were prepared. Cells were incubated with Ghost Dye Violet 510 (Tonbo Biosciences, San Diego, CA, USA) to stain dead cells. Cells were subsequently stained with surface antibodies for B- and T-cell marker proteins and by intracellular staining for key transcription factors. Fluorescent signals were collected with an LSRII or Fortessa flow cytometers. Antibodies for flow cytometry were obtained from BD Biosciences, BioLegend, eBioscience, Miltenyi Biotec, or R&D Systems. The following antibodies were used in flow analysis of B-cell subsets: B220 (clone RA3-6B2), CD21 (clone 7G6 or REA800), CD23 (clone B3B4), CD73 (clone eBioTY/11.8), CD80 (clone 16-10A1), CD138 (clone 281-2), FAS (clone Jo2), IgG1 (clone RMG1-1), PD-L2 (clone TY25), and peanut agglutinin (PNA, biotinylated from Vector Labs, Burlingame, CA, USA). The following antibodies were used in flow analysis of T-cell subsets: CD4 (clone GK1.5), CD8b (clone eBioH35-17.2), PD1 (clone 29F.1A12), and CXCR5 (clone L138D7). These antibodies were used for intracellular staining of key transcription factors regulating T helper cell subset differentiation: Tbet (clone 4B10), Foxp3 (clone FJK-16s), RORγT (clone B2D), GATA3 (clone L50-823), and BCL6 (clone 7D1). For isolation of skin-associated lymphocytes, depilated whole skin was harvested and floated on EDTA-free trypsin (Thermo Fisher) overnight at 4°C. The epidermis and dermis were then separated. The epidermal sheets were digested further in trypsin supplemented with 0.01% DNAse I (Millipore Sigma). The dermis was digested with 0.25 mg/mL Liberase (Millipore Sigma) and 1 mg/mL DNAse I (Millipore Sigma). The resulting cell suspensions were enriched for lymphocytes using Lymphoprep (Stemcell Technologies). Following enrichment, cells from the dermis and epidermis were combined and cultured overnight in the presence of 10 U/mL IL-2 (BioLegend). The following antibodies were used in flow analysis of skin-associated γδ T-cell populations: CD4 (clone GK1.5), CD8b (clone eBioH35-17.2), TCRγδ (clone GL3), Vγ5 (clone 536), and CCR6 (clone 29-2L17).

### *In vitro* differentiation of Tfh cells

Spleen and lymph nodes from mice were harvested, mechanically disrupted, and passed through a 70-μm filter. Naive CD4^+^ T cells from the sample were purified by magnetic bead separation using the CD4^+^CD62L^+^ isolation kit (Miltenyi Biotec). Cells were plated in 96-well plates coated with anti-CD3 antibody (5 μg/mL, BD, clone 145-2C11) at a density of 3 × 10^6^ cells/mL. Cells were plated in Tfh-conditioning media (RPMI complete media with 10% fetal bovine serum), 10 μg/mL anti-IL4 (BD Biosciences, clone 11B11), 10 μg/mL anti-IFNγ (BD Biosciences, clone XMG1.2), 10 μg/mL anti-TGFβ (R&D Systems, clone 1D11), 30 ng/mL IL6 (Shenandoah Biotechnology), and 50 ng/mL IL21 (Shenandoah Biotechnology) in the presence of soluble anti-CD28 (2 μg/mL, BD, clone 37.51). Polarized cells were harvested for Tfh phenotyping by flow cytometric analysis 4 days after plating.

For surface phenotype and transcription factor analysis, harvested cells were stained with Live/Dead Fixable Aqua dead cell stain (Thermo Scientific Fisher) to exclude dead cells. Cells were subsequently stained for surface markers (CD4, TCRβ, PD1, CXCR5) and then fixed and permeabilized using the Intracellular Fixation and Permeabilization Buffer Set (eBioscience). Fixed cells were stained with antibody to BLC6 (clone 7D1) or isotype control.

### ELISA

ELISA to detect total serum IgM and IgG and autoantibodies was performed as previously described (2). Serum IgE levels were detected using the BioLegend Mouse IgE ELISA Max Deluxe Set. Maxisorp 96 ELISA well plates were coated overnight with 10 μg/mL of antigens, and ELISA was performed as previously described (2).

### ELISpot

Single-cell suspensions were prepared from spleen and lymph nodes and plated on ELISpot plates to detect IgM- and IgG-secreting cells as previously described (2, 15). Spots were counted with an automated counter, and the number of antibody-secreting cells per million total cells plated was calculated.

### Immunostaining of Hep2 cells

Hep2 hepatoma cells on glass slides were incubated with 1:40 dilutions of mouse serum and subsequently with fluorescein isothiocyanate (FITC)-conjugated anti-mouse IgG. Staining was performed, and images were captured with a HELmed Integrated Optical System (HELIOS, AESKU Diagnostics).

### RNA-sequencing and analysis

Skin samples harvested from DKO and control mice were processed in TRIzol (Thermo Fisher), and bulk RNA was then purified. Purified RNA was then analyzed by QuBit/Quant-IT and Fragment Analyzer (Agilent) for quality control. cDNA library preparation was completed using TrueSeq RNA sample preparation kit (Illumina) followed by 50-bp single-end sequencing on an Illumina HiSeq 2500. Analysis of the raw reads using FASTQC v0.11.9 application was used as a quality control metric following sequencing.

The raw reads were then mapped to the GRCm38 (the mm10 reference mouse genome) using TopHat2 (34). The aligned reads were then quantified using featureCounts v1.5.3 to generate a raw count matrix. The raw count matrix was then processed in R to generate transcripts per million normalized expression values as previously described in Wagner et al. (35). Differential gene expression analysis was conducted comparing knockout samples to WT controls using DEseq2 v1.24.0 with genes being identified as statistically significantly differentially expressed with a log2fold change ≥1 and an false discovery rate (FDR) value of ≤0.1. RNA-sequencing data are available under GEO DataSets accession number GSE237696.

Upregulated and downregulated differentially expressed genes (DEGs) identified by the above analysis were then subjected to GO term enrichment analysis. DEGs were input into the online tool g;Profiler (36) to assess for gene ontology (GO) terms significantly enriched among the upregulated and downregulated genes.

### Analysis of susceptibility to *Staphylococcus* infection

To determine if DKO mice have spontaneous colonization of the skin with staphyloccal bacteria, we swabbed the ventral side of the neck and upper thorax and plated swabbed bacteria on mannitol-salt-agar (MSA) plates that specifically promote growth of staphylococcal species while suppressing the growth of other bacteria. Plates were incubated overnight at 37°C, and the colonies were subsequently counted. Genomic sequencing was used to confirm the species of staphylococcal bacteria. To determine if DKO mice are susceptible to exogenous *S. aureus* infection, a methicillin-resistant *Staphylococcus aureus* (MRSA) USA300 strain NRS-384 carrying a bioluminescent marker (the lux operon from *Photorhabdus luminescens*) was obtained from Dr. Roger Plaut at the Food and Drug Administration (FDA) (37). Bacteria were grown in tryptic soy agar to log phase, harvested, washed with PBS, and then resuspended at 200 million cells/mL. Mice to be infected were anesthetized followed by shaving the back skin and making three small topical cuts in the skin. Approximately 10 µL of resuspended bioluminescent *S. aureus* was introduced into the cuts. Mice were given buprenorphine to control pain. Infected mice were imaged on days 1, 3, 7, 14, and 21 post-infection using an *In vivo* imaging system (IVIS) imager that detects the bioluminescent signal. Luminescence values were normalized to the signal at day 1 to control for differences in cut size and depth between animals.

### Staining of ears for DETCs

Ears were harvested from mice and then treated with Nair to remove hair. The two leaflets of the ear were separated from the underlying cartilage. The ear leaflets were then floated on a 3.6% ammonium thiocyanate solution dermis side down to dissociate the dermis from the epidermis. The epidermal sheet was then peeled off and fixed using 4% formaldehyde. Normal goat serum supplemented with Triton X-100 was used to block and permeabilize the tissue followed by overnight staining using FITC anti-TCRγδ (clone GL3). The epidermal sheets were mounted on a slide using VECTASHIELD Antifade Mounting Medium containing 4’,6’-diamidino-2-phenylindole (DAPI), and the entire ear field was imaged using a Leica TCS SP8 confocal microscope system.

### Depletion of DETCs

WT and IL17RA KO animals were injected with 100 μg of Ultra-LEAF Purified Anti-mouse T cell receptor (TCR) Vγ5 antibody (clone 536) or Ultra-LEAF Purified Syrian Hamster IgG Isotype control antibody (clone SHG-1). For validation of depletion, epidermal sheets were harvested from ear leaflets as previously described (Jameson et al., 2004). Epidermal sheets were stained with FITC-anti-mouse TCRγδ (clone GL3) and TOPRO-3 Iodide (Thermo Fisher). For determination of DETC contribution to skin *S. aureus* infection immune responses, WT and IL17RA KO animals were injected with antibody as above and infected 2 days post-injection as described above. The burden of *S. aureus* was measured using an IVIS imager on days 1, 2, 3, 4, 5, 7, 10, 14, 21, 24, 28, 32, and 35 days post-infection with signal normalized to day 1 in order to control for cut size and depth variation.

### Statistics

One-way ANOVAs or nonparametric Kruskal–Wallis tests were performed followed by Tukey’s or Dunn’s multiple comparison test, respectively, except for the autoantibody ELISAs and IVIS imaging, where 2-way ANOVAs were used followed by a Bonferroni posttest. In all cases, error bars are standard error of the mean (SEM), and p < 0.05 was considered significant.

## Results

### Loss of IL-17 signaling promotes autoimmune disease in Ets1 knockout mice

**Figure 1:**
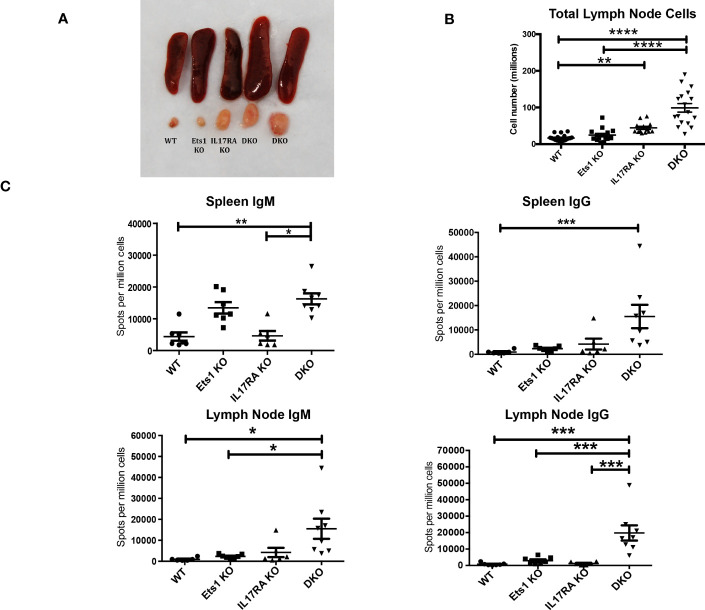
DKO mice have enlarged lymph nodes and increased plasma cells and serum antibodies. **(A)** Spleens and cervical lymph nodes harvested from wild-type, Ets1 KO, IL17RA KO, and DKO mice. **(B)** Total lymph node cells in mice of the indicated genotypes (n = 13–19 mice per genotype). **(C)** ELISpot quantification of IgM- and IgG-secreting plasma cells in spleen and lymph node of mice of the indicated genotypes (n = 6–8 mice per group). *p < 0.05, **p < 0.01, ***p < 0.001, ****p < 0.0001.

**Figure 2:**
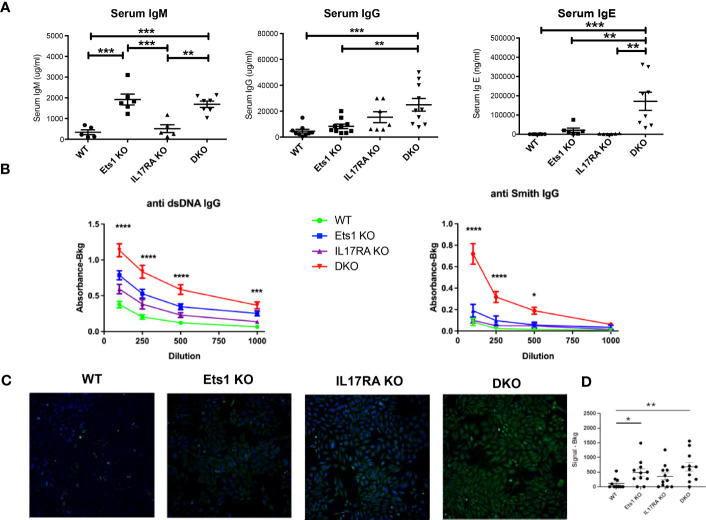
DKO mice have increased titers of autoantibodies. **(A)** ELISA quantification of total serum IgM, IgG, and IgE of mice of the indicated genotypes (n = 5–7 mice per group for IgM, n = 7–10 mice per group for IgG, n = 6–8 mice per group for IgE). **(B)** IgG autoantibodies to dsDNA and Smith antigen (Sm) in serum from wild-type (WT, n = 15), Ets1 KO (n = 12–13), IL17RA KO (n = 9–10), and DKO mice (n = 16–18). Asterisks represent statistical significance when comparing WT to DKO at each serum dilution. **(C)** A representative image of Hep2 cells stained with serum from mice of the indicated genotypes and with an FITC-conjugated anti-mouse IgG secondary antibody. **(D)** Quantification of the intensity of signal in Hep2 cell staining for sera derived from different genotypes of mice. N = 11 sera per genotype. *p < 0.05, **p < 0.01, ***p < 0.001, **** p < 0.0001.

In order to test the role of IL-17 in the autoimmune phenotype of Ets1-deficient mice, we crossed Ets1 KO mice with mice lacking the IL17RA to generate DKO mice. We analyzed DKO and control WT and single knockout mice at 3–6 months of age. While we anticipated that loss of IL-17 signaling might result in reduced immune cell activation and autoimmune phenotypes, we instead found that DKO mice had a stronger phenotype than Ets1 KO mice. This was shown by greatly enlarged peripheral lymph nodes (**Figures 1A, B**) and more modestly enlarged spleens in the DKO mice as compared to controls (**Figure 1A**, **Table 1**). ELISpot assays showed that there was a dramatic increase in antibody-secreting cells in DKO mice compared to Ets1 KO mice, especially IgG-secreting plasma cells in the lymph node (**Figure 1C**). Levels of serum IgM and IgE are higher in Ets1 KO than WT controls, whereas total serum IgG titers are not significantly increased (16, 17). Serum from DKO mice showed a similar increase in IgM as that found in Ets1 KO serum but had a much stronger increase in serum IgG and IgE (**Figure 2A**). On average, the serum IgE levels were >400-fold higher in DKO as compared to WT mice and ~5-fold higher in DKO as compared to Ets1 KO.

**Table 1:** Absolute numbers of B- and T-cell populations in the spleen and lymph node.

	Wild-type	Ets1^-/-^	IL17RA^-/-^	DKO
**Number of splenocytes (millions)**	50.0 ± 4.9	83.3 ± 12.7	40.9 ± 3.6	^***^ 111.4 ± 11.6
**Number of lymphocytes (millions)**	16.6 ± 1.9	24.7 ± 4.5	^**^ 44.3 ± 4.2	^****^ 99.0 ± 11.7
**Number of B220^+^ B cells (spleen)**	25.08 ± 3.3	41.7 ± 8.0	17.34 ± 2.3	^**^ 59.1 ± 5.9
**Number of B220^+^ B cells (lymph nodes)**	3.45 ± 0.9	10.49 ± 3.37	7.47 ± 2.27	^****^ 47.56 ± 7.34
**Percent of B220^+^ B cells (spleen)**	42.64 ± 3.12	46.42 ± 3.35	42.16 ± 3.62	50.6 ± 2.07
**Percent of B220^+^ B cells (lymph nodes)**	17.36 ± 2.05	^*^34.58 ± 4.09	14.88 ± 3.12	^***^43.64 ± 2.49
**Number of CD4^+^ T cells (spleen)**	11.8 ± 1.43	19.42 ± 3.01	8.49 ± 0.99	^*^ 20.08 ± 2.33
**Number of CD4^+^ T cells (lymph nodes)**	6.31 ± 0.82	7.97 ± 1.30	^***^ 18.27 ± 2.11	^****^ 21.17 ± 2.40
**Percent of CD4^+^ T cells (spleen)**	22.88 ± 2.65	23.92 ± 1.33	22.23 ± 2.01	18.85 ± 1.08
**Percent of CD4^+^ T cells (lymph nodes)**	39.11 ± 3.03	36.38 ± 3.56	41.93 ± 3.21	^***^22.91 ± 1.40

*p < 0.05, **p < 0.01, ***p < 0.001, ****p < 0.0001 (when compared to wild-type controls).

DKO mice had higher titers of autoantibodies against dsDNA and Smith antigen (Sm) than Ets1 KO mice (**Figure 2B**). Hep2 cell staining demonstrated that Ets1 KO and DKO mice produce anti-nuclear and anti-cytoplasmic IgG autoantibodies (**Figure 2C**). Unexpectedly, we also found that IL17RA KO mice produce autoantibodies as well. The intensity of Hep2 staining was in general stronger using serum from DKO mice than Ets1 KO or IL17RA KO, indicating increased levels of autoantibodies in DKO mice (**Figure 2D**).

### DKO mice have more activated B and T cells than Ets1 KO mice

**Figure 3:**
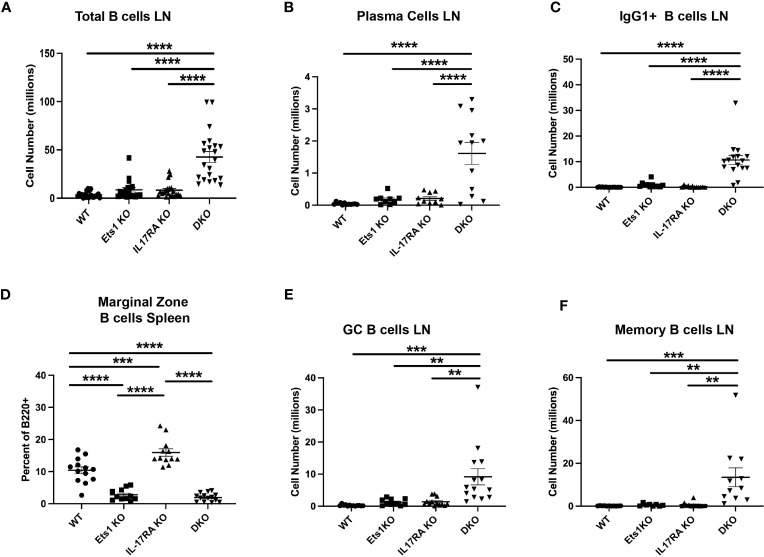
DKO mice have more activated and class-switched B cells. **(A)** Total B220^+^ B cells in lymph nodes of mice of the indicated genotypes (n = 18–21 mice per genotype). **(B)** Quantification of the numbers of plasma cells (B220-low CD138^+^) (n = 9–12 mice per genotype) and **(C)** IgG1^+^ B cells (B220^+^IgG1^+^) (n = 11–16 mice per genotype) in lymph nodes. **(D)** Percent of CD21^hi^ CD23^low^ marginal zone B cells among total B220^+^ B cells in the spleen (n = 11–15 mice per genotype). **(E)** Quantification of germinal center B cells (B220^+^Fas^+^PNA^+^) (n = 10–14 mice per genotype) and **(F)** memory phenotype B cells (B220^+^CD80^+^PDL2^+^) (n = 8–11 mice per genotype) in the lymph nodes of mice of the indicated genotypes. * **p < 0.01, ***p < 0.001, ****p < 0.0001.

To further explore the phenotype of DKO mice, we analyzed B- and T-cell differentiation status using flow cytometry. DKO mice have increased total B-cell numbers in the spleen and lymph nodes compared to controls (**Figure 3A**, **Table 1**). Similarly, DKO mice showed a striking increase in the percentages and numbers of plasma cells and IgG1 class-switched B cells compared to Ets1 KO mice (**Figures 3B, C**; **Supplementary Figures S1, S2**). Like Ets1 KO mice, DKO showed a strong reduction of marginal zone B cells in the spleen (**Figure 3D**). We found that there was an increase in the numbers of germinal center B cells (B220^+^PNA^+^Fas^+^) in the lymph nodes and an increase in the numbers of B cells with a memory phenotype (B220^+^CD80^+^PDL2^+^) in both the spleen and the lymph nodes of DKO mice as compared to controls (**Figures 3E, F**; **Supplementary Figures S3, S4**). These results indicate that DKO mice have a greatly expanded germinal center response and an overall high level of B-cell activation.

**Figure 4:**
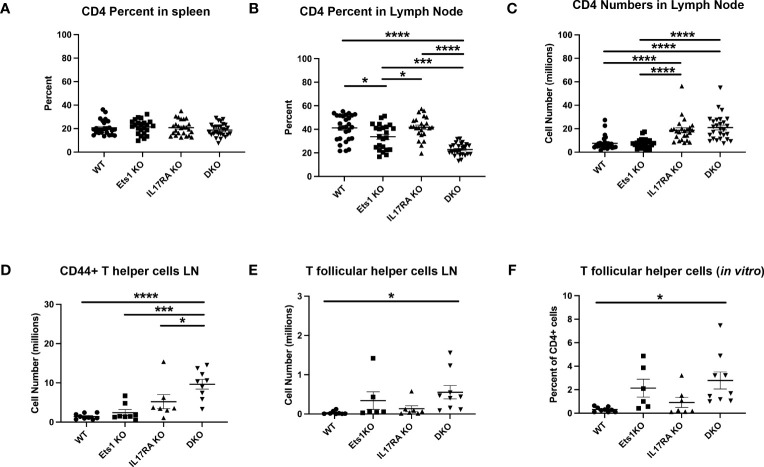
DKO mice have more activated T cells and increased numbers of Tfh. **(A, B)** Percent of CD4^+^ T cells in the spleen and lymph nodes of mice (n = 22–27 mice per genotype). **(C)** Total numbers of CD4^+^ T cells in the lymph nodes of mice (n = 22–27 per genotype). **(D)** Quantification of the numbers of CD4^+^ T cells with an activated/memory phenotype in the lymph nodes (CD4^+^CD44^+^) (n = 7–9 mice per genotype). **(E)** Quantification of CD4^+^ T cells with a Tfh phenotype (PD1^+^CXCR5^+^) in the lymph nodes (n = 6–9 mice per genotype). **(F)** Quantification of the percentage of Tfh cells (PD1^+^CXCR5^+^ICOS^+^BCL6^+^) among total live CD4^+^ cells in *in vitro* cultures (n = 6–9 mice per genotype). *p < 0.05, ***p < 0.001, ****p < 0.0001.

In the T-cell compartment, we found that while the overall percentage of CD4^+^ T cells in the spleen was similar in all genotypes (**Figure 4A**), the percentage of CD4^+^ T cells in the lymph nodes of DKO mice was reduced (**Figure 4B**, **Table 1**). However, because both IL17RA KO and DKO lymph nodes are larger than those of control mice, the total number of CD4^+^ T cells was in fact higher in IL17RA KO and DKO mice than in WT or Ets1 KO controls (**Figure 4C**, **Table 1**). Many CD4^+^ T cells in the lymph nodes of IL17RA KO and especially DKO mice expressed CD44, a marker of an activated or memory phenotype (**Figure 4D**). Furthermore, there was an increased number of cells with a Tfh phenotype (CD4^+^ PD1-hi CXCR5-hi BCL6^+^) in Ets1 KO and DKO spleen and lymph nodes (**Figure 4E**, **Supplementary Figure S5**). To further examine the propensity of DKO T cells to become Tfh, we isolated naive CD4^+^ T cells from the spleens and lymph nodes of DKO and control mice and stimulated them *in vitro* under conditions that promote Tfh differentiation. CD4^+^ T cells from Ets1 KO and DKO mice showed increased differentiation to PD1^+^ CXCR5^+^ ICOS^+^ BCL6^+^ Tfh cells (**Figure 4F**, **Supplementary Figure S6**).

We also examined the expression of Foxp3 in CD4^+^ T cells to assess the numbers of Treg cells. A previous report had indicated that there was a 3–4-fold reduction in the percentages of Foxp3^+^ Tregs in the spleens of Ets1 KO mice (17). In our studies, we found that the number of Foxp3^+^ Tregs was similar in the spleens and lymph nodes of WT, Ets1 KO, and IL17RA KO mice and was elevated in DKO mice (**Supplementary Figures S7A, B**). However, similar to a previous study (17), we did find that the intensity of Foxp3 staining was lower in CD4^+^ T cells from mice lacking Ets1, especially in lymph node T cells (**Supplementary Figure S7C**). We also analyzed staining of Tbet, GATA3, and Rorγt in spleen and lymph node cells to determine if there was spontaneous differentiation of CD4^+^ T cells to Th1, Th2, or Th17 fates. The total number of CD4^+^ T cells that stained with GATA3 and RORγt antibodies was elevated in DKO mice (**Supplementary Figures S7C, D**).

### DKO mice have increased susceptibility to staphylococcal skin infections

**Figure 5:**
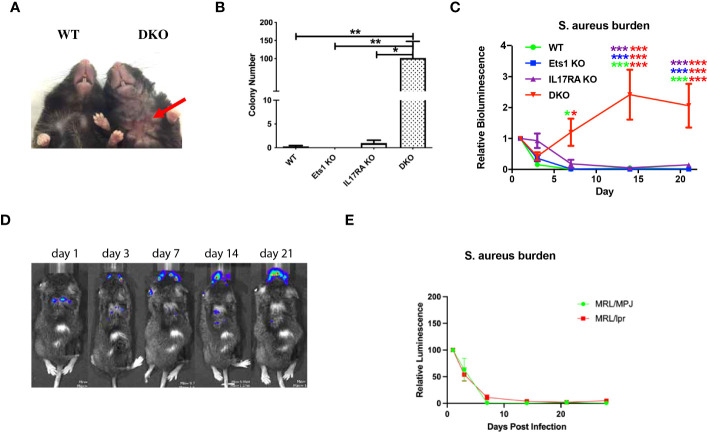
DKO mice are susceptible to skin infections with *S. aureus*. **(A)** Representative skin lesion on the ventral side of the neck of a DKO mouse (red arrow indicates lesion) compared to an unaffected wild-type (WT) control. **(B)** Quantification of the recovery of *S. xylosus* colonies from swabbing the ventral neck skin of mice of the indicated genotypes (n = 4–7 per genotype). **(C)** Quantification of IVIS imaging of 4-month-old DKO and control mice infected with bioluminescent *S. aureus*. Mice were imaged on days 1, 3, 7, 14, and 21 post-infection, and signals were normalized to those on day 1. Asterisks are color coded for the strains compared (using the colors in the legend), and the number of asterisks of any one color above a particular point on the curve represents the significance of the difference at that time point, and the two colors on each line of asterisks represent the two genotypes compared (n = 5–7 per genotype). **(D)** Representative IVIS images of a DKO mouse showing shifting of the bioluminescent signal from the back, where the infection was initiated, to the face. **(E)** Quantification of IVIS imaging of 4-month-old autoimmune MRL/lpr mice and non-autoimmune control MRL/MpJ mice. N = 6 MRL/MpJ and 13 MRL/lpr mice. *p < 0.05, **p < 0.01, ***p < 0.001.

By 6 months of age, most DKO mice began to develop skin dermatitis and lesions, often on the ventral surface of the neck (**Figure 5A**). Such lesions were not found on Ets1 KO mice in our colony and were found at a lower rate and lesser severity in IL17RA KO mice. RNA-sequencing experiments using RNA isolated from the skin of DKO and control mice showed upregulation of many pathways associated with an inflammatory immune response, including the response to *S. aureus* infection (**Supplementary Figure S8A**). Indeed, many cytokines and chemokines involved in the skin immune response were highly overexpressed in the skin of DKO mice as compared to control mice (**Supplementary Figure S8B**).

Mice lacking IL17RA are known to have a defect in clearing *S. aureus* from the skin (27). Given this observation and the results of RNA-sequencing, we sought to determine whether staphylococcal bacteria could be recovered from the skin of DKO. Swabbing the ventral neck of mice resulted in the recovery of high levels of staphylococcal bacteria from the skins of 100% of DKO mice tested (whether or not they showed visible lesions), while staph was cultured from only two of six IL17RA KO mice tested using this technique. The level of staph bacteria recovered from IL17RA KO mice was also lower than that found in DKO mice (**Figure 5B**). Genomic sequencing showed that recovered bacteria were *Staphylococcus xylosus* (**Supplementary Figure S8C**), a staph species prominent on mouse skin (38–40) and a frequent cause of skin infections in mice (41–45).

To determine whether DKO mice have an impaired immune response to staphylococcal bacteria, we experimentally infected cuts in the skin of DKO and control mice with a bioluminescent strain of *S. aureus* (37). *S. aureus* and *S. xylosus* are closely related staphylococcal bacteria, and both are common colonizers of the skin. Because bioluminescent *S. xylosus* is not available, examining DKO responses to *S. aureus* was used to determine if there is a generalized impairment of anti-staphylococcal immunity in these mice. We monitored the bioluminescent signal at days 1, 3, 7, 14, and 21 after infection. In this assay, Ets1 KO mice did not show any increased susceptibility and cleared the infection with kinetics similar to WT mice (**Figure 5D**). As previously reported, mice lacking IL17RA showed a susceptibility to *S. aureus* with initially delayed clearance (**Figure 5C**) but were able to fully clear the infection by day 21 post-infection. On the other hand, DKO mice initially started to clear the infection and on day 3 actually showed a signal that was lower than that of IL17RA KO mice and comparable to that of WT and Ets1 KO controls. However, by day 7, the infectious signal in DKO mice increased dramatically and remained elevated until day 21 and beyond (**Figure 5C**). Additionally, although the initial infections were made in the skin of the upper back, in some mice, the infection in DKO mice spread to the face/neck area (**Figure 5D**). We considered whether the susceptibility to skin infection by staphylococcal bacteria might be due to autoimmune-mediated skin damage, given the strongly enhanced autoimmunity in DKO mice. To test whether autoimmune skin damage promotes susceptibility to staph infection, we tested autoimmune MRL/lpr as compared to control non-autoimmune MRL/MpJ mice in the bioluminescent staph infection model. MRL/lpr mice are known to have autoimmune-mediated skin damage, with lesions similar to human cutaneous lupus (46, 47). Despite this, we found that MRL/lpr mice could clear exogenous skin infections with *S. aureus* with the same kinetics as control MRL/MpJ mice (**Figure 5E**). Thus, the presence of autoimmune skin damage *per se* does not lead to a defect in staph clearance.

### Alterations in γδ T-cell subsets in mice lacking Ets1

**Figure 6:**
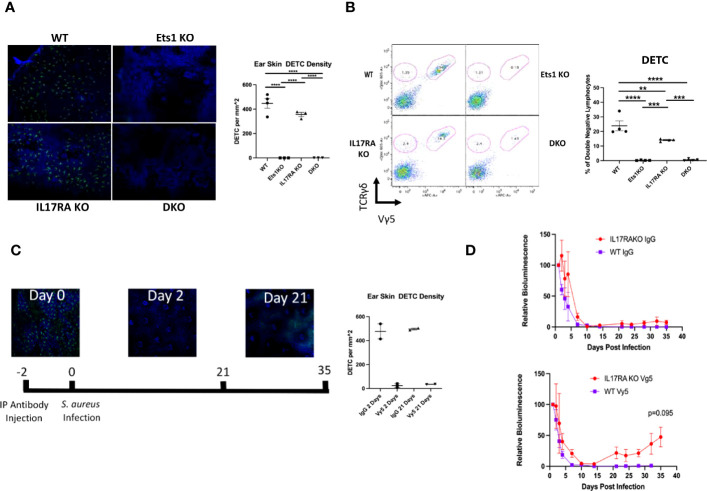
Mice lacking Ets1 lack DETCs in the skin, and DETCs are required to clear skin staphylococcal infections. **(A)** Immunostaining of γδ T cells in the skin of the ear of DKO and control mice. Green staining is for the γδ TCR, and blue staining is nuclei. The graph shows quantification of the number of DETCs per mm^2^ of tissue. N = 3–4 mice per genotype. **(B)** Representative flow cytometry plot for γδ T cells in the skin of mice. Plots are gated on CD4^-^CD8^-^ lymphocytes and shown is staining of TCR γδ versus TCR Vγ5 (the canonical TCR of DETCs). DETCs are double positive for both markers, and other γδ T-cell subsets are positive only for TCR γδ. Graph on the right shows quantification of TCR Vγ5^+^ lymphocytes. N = 4 mice of each genotype. **(C)** Immunostaining of γδ TCR^+^ cells (green) and nuclei (blue) before and after Vγ5^+^ cell depletion. Shown at the bottom is the timeline of depletion and infection. Graph shows quantification of depletion at day 2 and day 21 post-injection of depleting antibody. N = 2 mice per time point. **(D)** Quantification of IVIS imaging of 4-month-old DKO and control mice injected with either control IgG (left panel) or anti-TCR Vγ5 antibody (right panel) and then infected with bioluminescent *S. aureus*. N = 2–7 mice per genotype. **p < 0.01, ***p < 0.001, ****p < 0.0001.

Mice completely lacking γδ T cells have been shown to have a defect in the clearance of staph infections from the skin, while mice lacking αβ T cells were able to clear (27). We found that DKO mice and Ets1 KO mice lack γδ TCR^+^ cells in the epidermis (**Figure 6A**), while IL17RA KO and WT mice have these cells. These results were verified using flow cytometry to identify Vγ5^+^ and Vγ5^-^ γδ T cells in the skin. As shown in **Figure 6B**, DKO and Ets1 KO mice lack Vγ5^+^ DETC cells in the skin, while IL17RA KO and WT mice have such cells. On the other hand, all genotypes of mice had Vγ5^-^ γδ T cells in the skin, and these tended to be slightly increased in the skin of DKO and IL17RA KO mice.

The specific role of DETCs in responding to skin staph infection is unknown. To determine if DETCs might be required for clearance of staph infection, we depleted DETCs using anti-Vγ5 antibody (**Figure 6C**). Since DETCs are the only γδ T cells that express the Vγ5 receptor, the depletion is specific to DETCs and does not affect other γδ T-cell subsets. DETCs were depleted from both WT and IL17RA KO mice, and the mice were subsequently infected with bioluminescent staph and the infection followed with IVIS imaging. As shown in **Figure 6D**, IL17RA KO mice that had DETCs depleted initially showed normal kinetics of clearance. However, by day 20 post-infection, the bioluminescent signal in depleted IL17RA KO mice began to increase. This was not seen in IL17RA KO mice injected with isotype control Ab or in WT mice injected with either isotype control or Vγ5 antibody. Similar to what we saw in a subset of DKO mice, we found that the infection in some IL17RA KO depleted for DETCs shifted from the initial site of infection on the upper back to the face region (not shown). These results indicate that DETCs cooperate with pathways induced by IL-17 signaling to mediate complete clearance of staph infections from the skin.

## Discussion

In order to explore the potential contribution of the cytokine IL-17 to the autoimmune phenotype of Ets1 KO mice, we generated DKO mice lacking both Ets1 and the IL-17 receptor subunit IL17RA. IL17RA KO mice are resistant to a number of autoimmune diseases including experimental autoimmune encephalomyelitis (48), collagen-induced arthritis (49), autoimmune glomerulonephritis (50), and spontaneous lupus in the BDX2 mouse model (51). This and other data support a pro-inflammatory role for signaling through IL17RA. However, a number of observations also support an anti-inflammatory role for IL-17. For instance, in autoimmune uveitis, IL-17 has been found to be protective rather than pathogenic (52). Similarly, in sodium dextran sulfate-induced colitis, IL-17 is protective (53). In autoimmune B6.lpr mice, deletion of IL17RA leads to enhanced lymphoproliferation, although levels of anti-DNA autoantibodies are not enhanced (54). In the current study, we demonstrate that DKO mice lacking both Ets1 and IL17RA have worse autoimmune symptoms than Ets1 KO mice, suggesting that IL-17 signaling is required to limit autoimmune responses in Ets1 KO mice.

The worsened autoimmune response in DKO mice is particularly evident in the extremely enlarged skin-draining lymph nodes, which contain dramatically increased numbers and percentages of germinal center B cells, class-switched B cells, memory B cells, and plasma cells. Class switching was predominantly to the IgG1 and IgE isotypes, which is classically promoted by the cytokine IL-4. Ets1 KO mice are known to have increased numbers of Tfh cells that secrete IL-4 (Tfh2 cells) (22). In keeping with this, we found increased numbers of T cells with a Tfh phenotype and increased numbers of T cells that express GATA3 in spleens and lymph nodes of DKO mice. We found that there was a 2–3-fold increase in the percentage of CD4^+^ Treg cells in DKO mice, whereas we did not find a change in the percentage of Treg cells in Ets1 KO mice. These data are contradictory to a previous report that found a 3–4-fold decrease in Foxp3^+^ Tregs in Ets1 KO mice (17). The difference between our study and the prior one may be a result of different Ets1 KO alleles used or a difference in genetic background, age, or sex of the mice analyzed. Despite the fact that there are significant increases in the numbers of Treg cells in DKO mice, these cells likely have impaired function, since they express lower than normal levels of Foxp3. Indeed, a previous study has shown that Treg cells lacking Ets1 have reduced suppressive activity toward effector T cells (17).

In addition to the enhanced autoimmune phenotype detected in DKO mice, these mice also displayed a striking susceptibility to bacterial skin infections. DKO mice spontaneously develop skin lesions colonized by *S. xylosus*. Furthermore, 4-month-old DKO mice experimentally infected with bioluminescent *S. aureus* showed an inability to clear the infection. Previously, Ets1 KO mice on a C57BL/6 genetic background were shown to develop skin dermatitis when housed under conventional non-SPF conditions (55). In SPF conditions, Ets1 KO mice showed milder skin symptoms, characterized by increased ear thickness and edema (55). In our studies, we analyzed Ets1 KO carrying the same KO allele as those described by Lee et al. (55) However, our Ets1 KO mice and DKO mice were maintained on a mixed C57BL/6 × 129Sv genetic background due to higher viability of the mice on a mixed background. All of our mice were housed in SPF conditions, and Ets1 KO animals in our colony show very low levels of skin dermatitis that is not different from that found occasionally in WT mice in the same colony. On the other hand, IL17RA KO mice do develop skin dermatitis at increased frequency, as has been described previously (56). When compared to IL17RA KO mice, DKO mice develop more severe and extensive skin lesions and at a higher penetrance, indicating cooperation between Ets1 and IL17RA in regulating susceptibility to such infections.

Given the previously established importance of γδ T cells in controlling *S. aureus* skin infections (27), we examined γδ T-cell populations in the skin of DKO mice. We found that both Ets1 KO mice and DKO mice completely lack the DETC subset of γδ T cells. DETCs are located within the epidermal layer and intercalate between the keratinocytes with dendritic-like cellular projections. They have been shown to have an important role in skin wound healing by the production of epithelial growth factors (Fgf7, Fgf10, and Igf1), inflammatory cytokines [IL-2 and severe combined immunodeficient (IFNγ)], and chemokines (Ccl3, Ccl4, Ccl5, and Xcl1) (57). DETCs are also important for homeostatic maintenance of skin in the absence of wounding by producing IGF-1 that inhibits keratinocyte apoptosis (58). Interestingly, Ets1 KO mice have previously been shown to have a defect in skin wound healing (59). This defect was ascribed to impaired wound angiogenesis in the absence of Ets1 but may also be in part due to a lack of DETCs in these mice.

Mice completely lacking all γδ T-cell subsets show an impaired ability to clear skin infections with *S. aureus* (27, 60). Various subsets of γδ T cells are found in the skin, including Vγ5^+^ DETCs and Vγ4^+^ and Vγ6^+^ cells that secrete IL-17 (61, 62). γδ T cells carrying an invariant Vγ6 chain have been shown to expand upon *S. aureus* infection, while DETCs and other γδ T-cell subsets are not expanded (63). That result suggests that invariant Vγ6^+^ cells may be important to skin immune responses to *S. aureus* infection. Vγ6^+^ cells make IL-17, and both IL-17 and the IL-17 receptor are required for clearance of skin *S. aureus* infections (27). Our results suggest that DETCs are also one of the subsets of γδ T cells that contribute to anti-staph immune responses, since IL17RA KO mice depleted of DETCs showed enhanced susceptibility to infection. This role of DETCs was only revealed in mice lacking IL17RA, but not in WT mice, which is consistent with the lack of observed susceptibility to *S. aureus* skin infection in Ets1 KO animals. While depletion of DETCs in IL17RA mice leads to failure to clear the staph infection, the kinetics of this response differ somewhat from those of DKO mice (compare **Figures 5C**, **6D**). This observation hints that there may be additional defects in the skin or in the immune response to staphylococcal infection in DKO mice that are not mimicked by depleting DETCs from IL17RA KO mice, and future studies will explore this possibility.

DETCs may contribute to anti-staph immunity by promoting keratinocyte proliferation and wound healing, thus recreating the skin barrier and excluding entry of bacteria from the surface. The Vγ6^+^ cells that expand in response to staph infection carry a canonical invariant TCR chain with CDR3 regions encoding the amino acid sequence CACWDSSGFHKVF (63). The invariant TCR of DETCs has the same amino acid sequence in the Vγ5 CDR3 region. The TCR of DETCs has been shown to recognize a self-ligand expressed by wounded or stressed keratinocytes (64). Invariant Vγ6^+^ cells may then respond to the same or similar TCR ligands given the identical sequence of the TCR Vγ CDR3 region. Therefore, it appears that multiple γδ T-cell subsets may be involved in anti-staph skin immunity. The DETC subset may contribute by promoting wound healing to prevent the invasion of bacteria from the skin surface, while the Vγ6^+^ subset may contribute by producing IL-17 that induces production of chemokines (attracting neutrophils) and antimicrobial peptides (directly killing bacteria).

While human skin lacks a direct counterpart of DETCs, there are γδ T cells in human skin and they have been implicated in wound-healing responses (65). These cells may contribute to the clearance of *S. aureus* skin infections by promoting wound closure. Additional data also support a potential role for human γδ T cells in the response to *S. aureus*. In interferon (SCID) mice with a humanized immune system, treatment with pamidronate, a ligand for the Vγ2Vδ2 TCR, resulted in increased clearance of intraperitoneal bacterial infections, including *S. aureus* infection (66). Recent data also show a role for human γδ T cells in promoting dendritic cell activation in the context of *S. aureus* infection and thereby resulting in increased CD4^+^ T-cell activation (67). *S. aureus*-infected antigen presenting cells (APCs) can also stimulate human γδ T-cell production of IFN-γ (68).

A number of studies point to potential relevance of *S. aureus* skin infections in lupus patients. When the skin microbiome of lupus patients was compared to that of healthy controls, lupus patients showed enhanced colonization by *S. aureus* (69). In another study focusing on cutaneous lesions in lupus patients, *S. aureus* was recovered from approximately half of the lupus skin lesions, while it was not recovered from skin lesions of patients with psoriasis (70). Lupus patients whose skin lesions were colonized by *S. aureus* tended to have worse Cutaneous Lupus Erythematosus Disease Area and Severity Index (CLASI) scores. In addition to colonizing the skin, *S. aureus* can also be carried nasally. While lupus patients were not found to have higher rates of nasal carriage than control subjects, those lupus patients who did have nasal *S. aureus* colonization had elevated anti-dsDNA, anti-RNP, anti-SSA, and anti-SSB autoantibody titers and increased rates of kidney disease, furthering a link between *S. aureus* and lupus (71). In another study, nasal carriage of *S. aureus* was found to be associated with hypocomplementemia and with the occurrence of disease flares during the time frame of the study (72). Together, these results implicate *S. aureus* in the induction and progression of human lupus autoimmunity. Colonization with *S. aureus* may trigger increased immune cell activation and lead to disease flares in patients with SLE. Recently, *S. aureus* skin colonization was shown to promote the development of lupus-like disease in mice with an epithelial cell–specific deletion of IκBζ (Nfkbiz^ΔK5^) (73). Similarly, staphylococcal skin colonization in DKO mice may trigger enhanced immune activation, and this may account for the strongly enhanced Tfh, germinal center, and plasma cell responses. This is consistent with the dramatic enlargement of skin-draining lymph nodes, while interior lymph nodes and the spleen showed a less dramatic enlargement in DKO mice. In summary, the results presented in this report suggest two important conclusions: 1) that DETCs function along with pathways triggered by IL-17 signaling to induce protective skin immune responses to staphylococcal skin infection and 2) that chronic skin infection by staphylococcal bacteria may trigger enhanced activation of immune cells in the draining lymph nodes leading to the intensification of an underlying autoimmune response.

## Data availability statement

The datasets presented within this study are available online. RNA-sequencing data is available on the Gene Expression Omnibus (https://www.ncbi.nlm.nih.gov/geo/) with the following accession number GSE237696.

## Ethics statement

The animal study was approved by Roswell Park Cancer Center IACUC. The study was conducted in accordance with the local legislation and institutional requirements.

## Author contributions

MB, AS, WL, RJ, AS, ML, and LG-S performed the experiments. MB, AS, WL, RJ, LM, SS, EW, and LG-S designed and interpreted the experiments. MB, AS, and LG-S wrote the article. All authors read, edited, and approved the final version.
