# Supplementary material for: Ets1 and IL17RA cooperate to regulate autoimmune responses and skin immunity to Staphylococcus aureus
Source: Front Immunol. 2023 Aug 23;14:1208200. doi: 10.3389/fimmu.2023.1208200 (PMC10486983; doi:10.3389/fimmu.2023.1208200)

Supplemental Figure 1

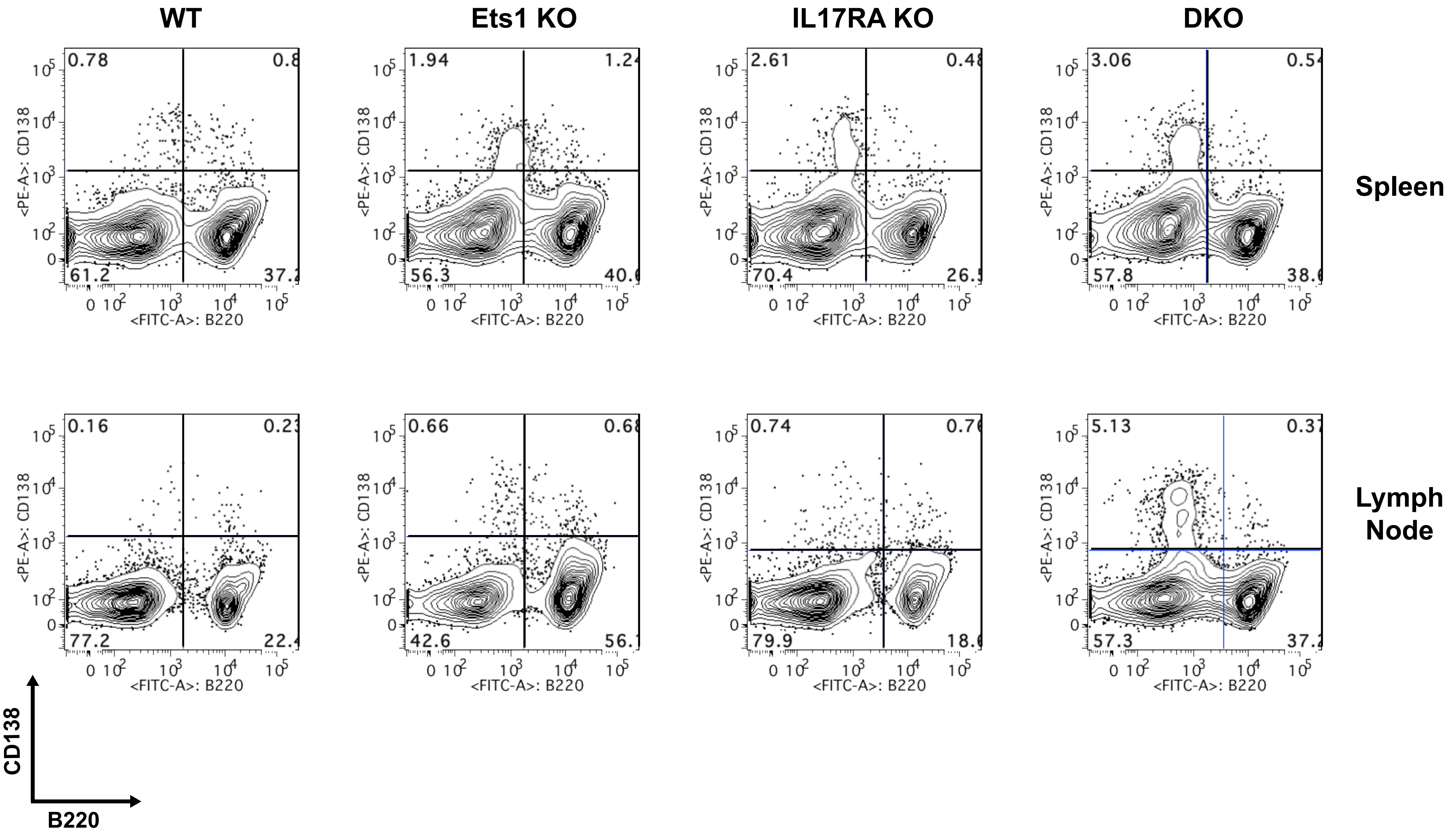

Supplemental Figure 2

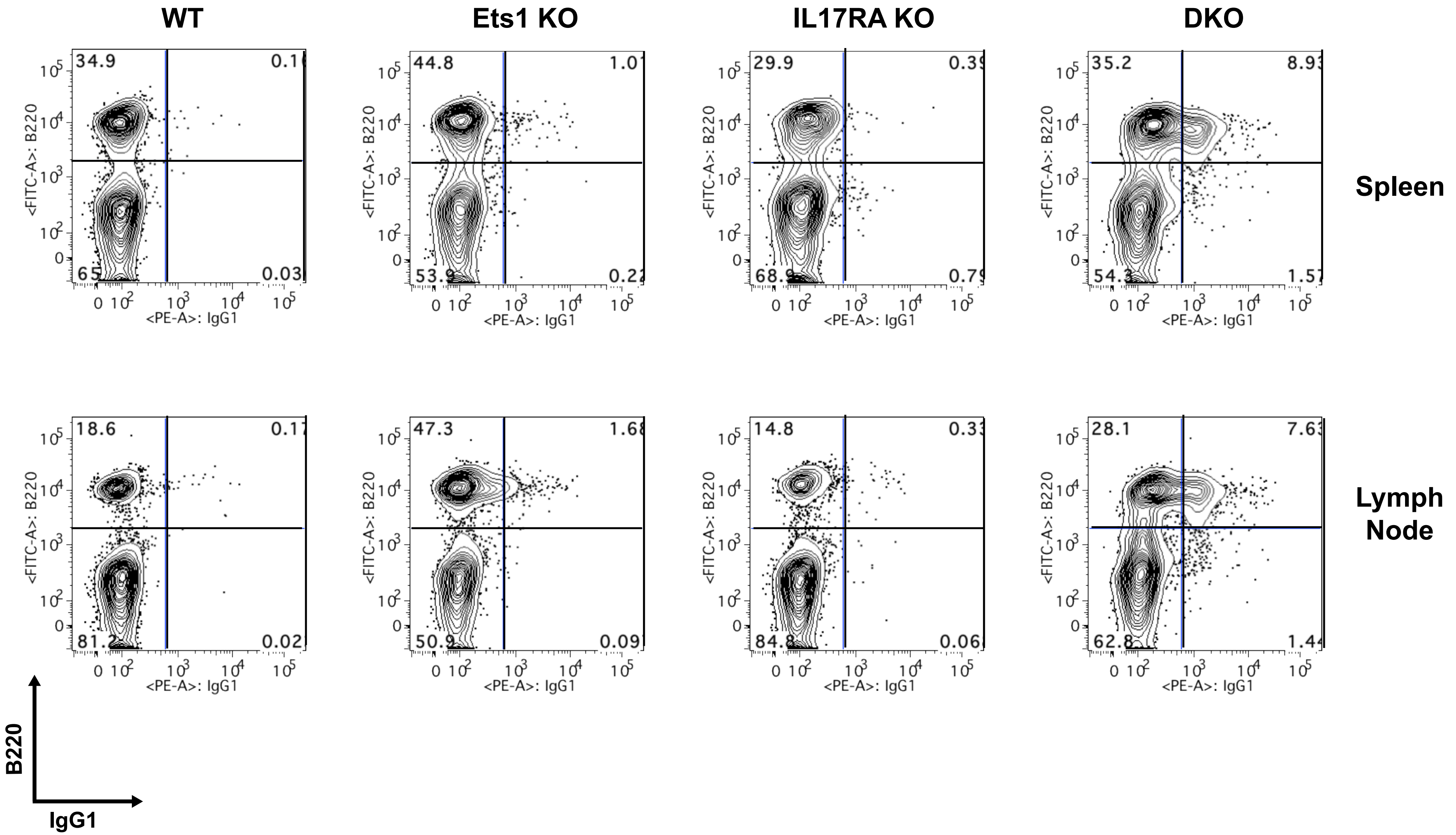

Supplemental Figure 3

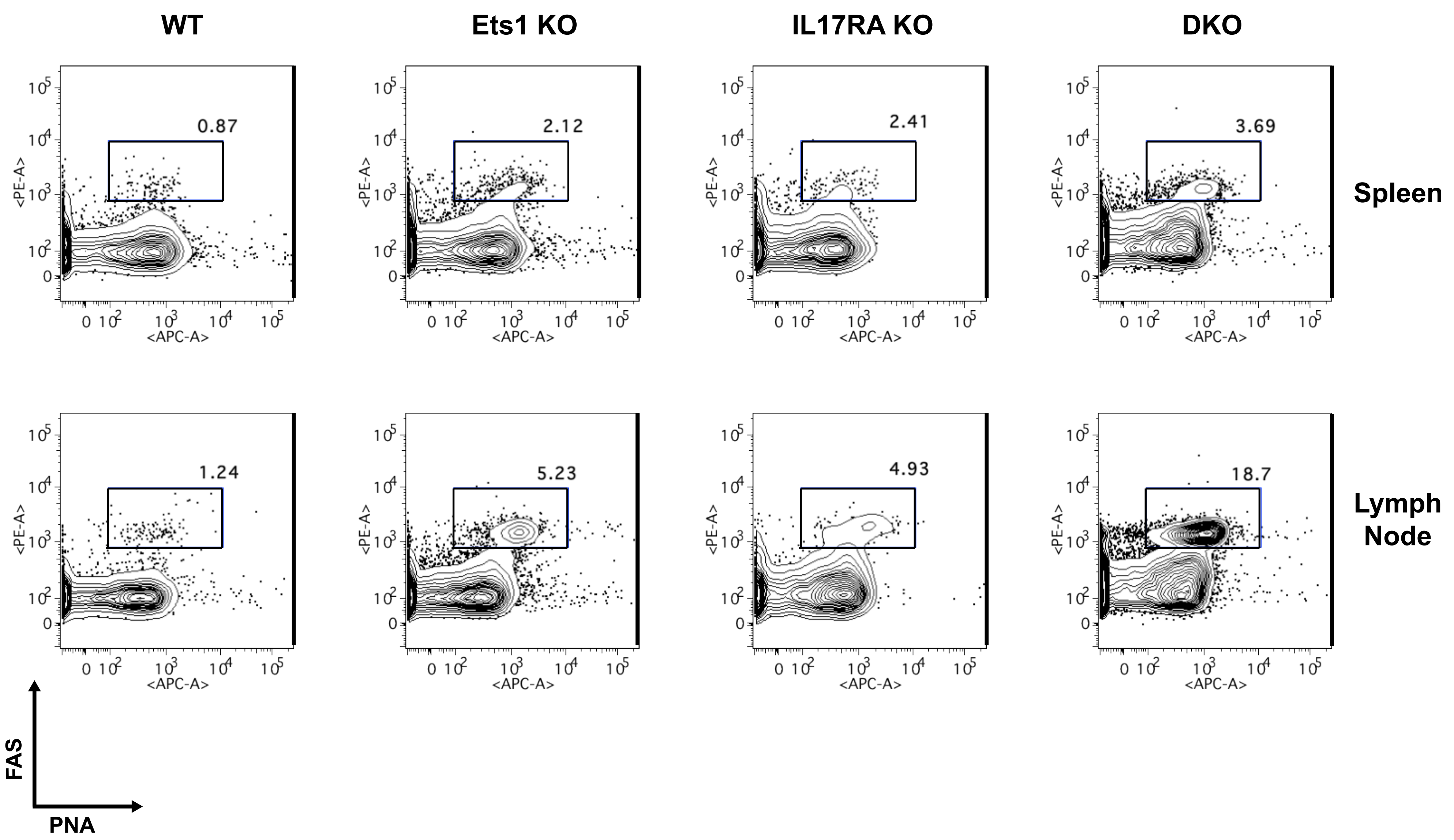

Supplemental Figure 4

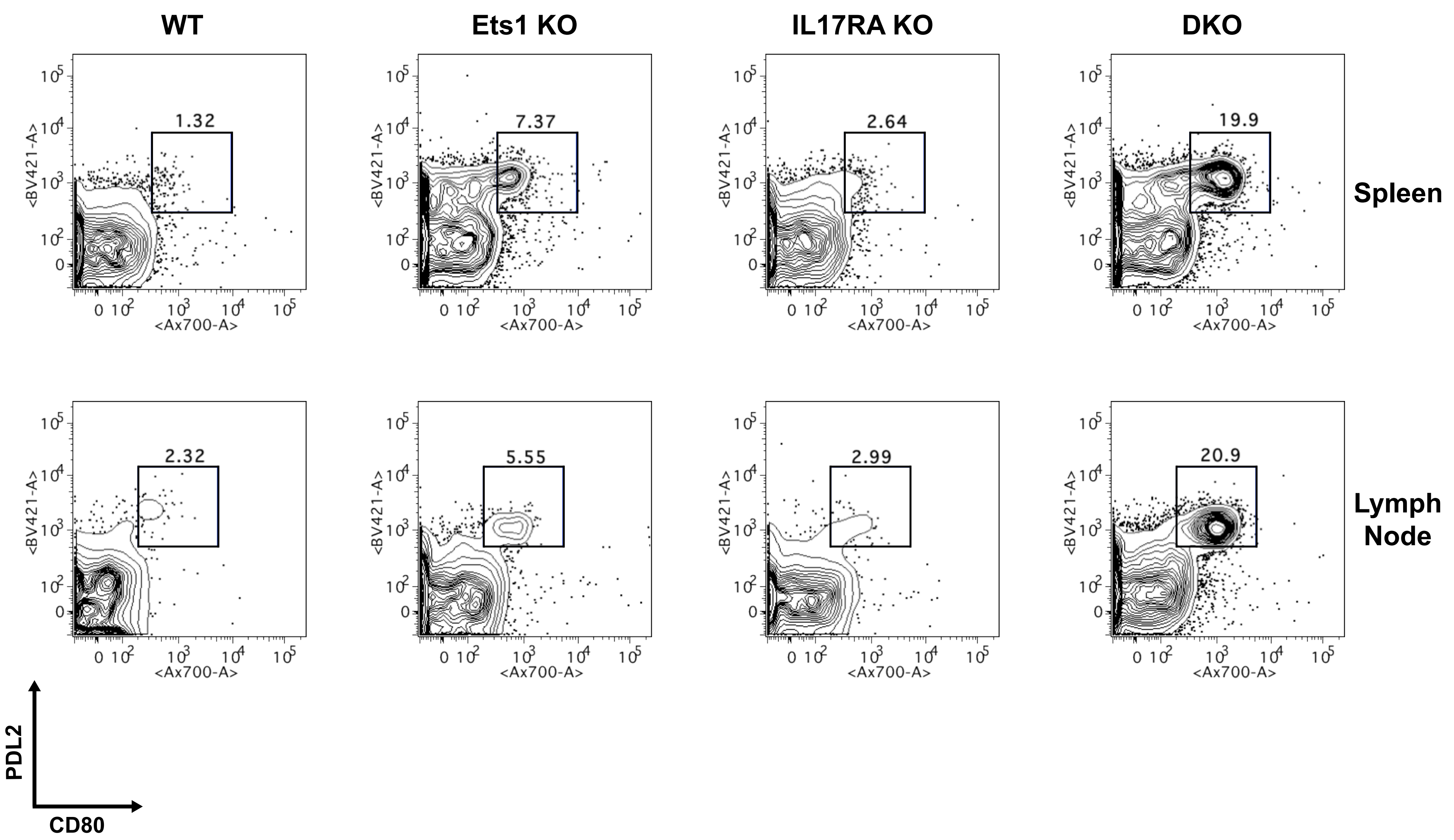

Supplemental Figure 5

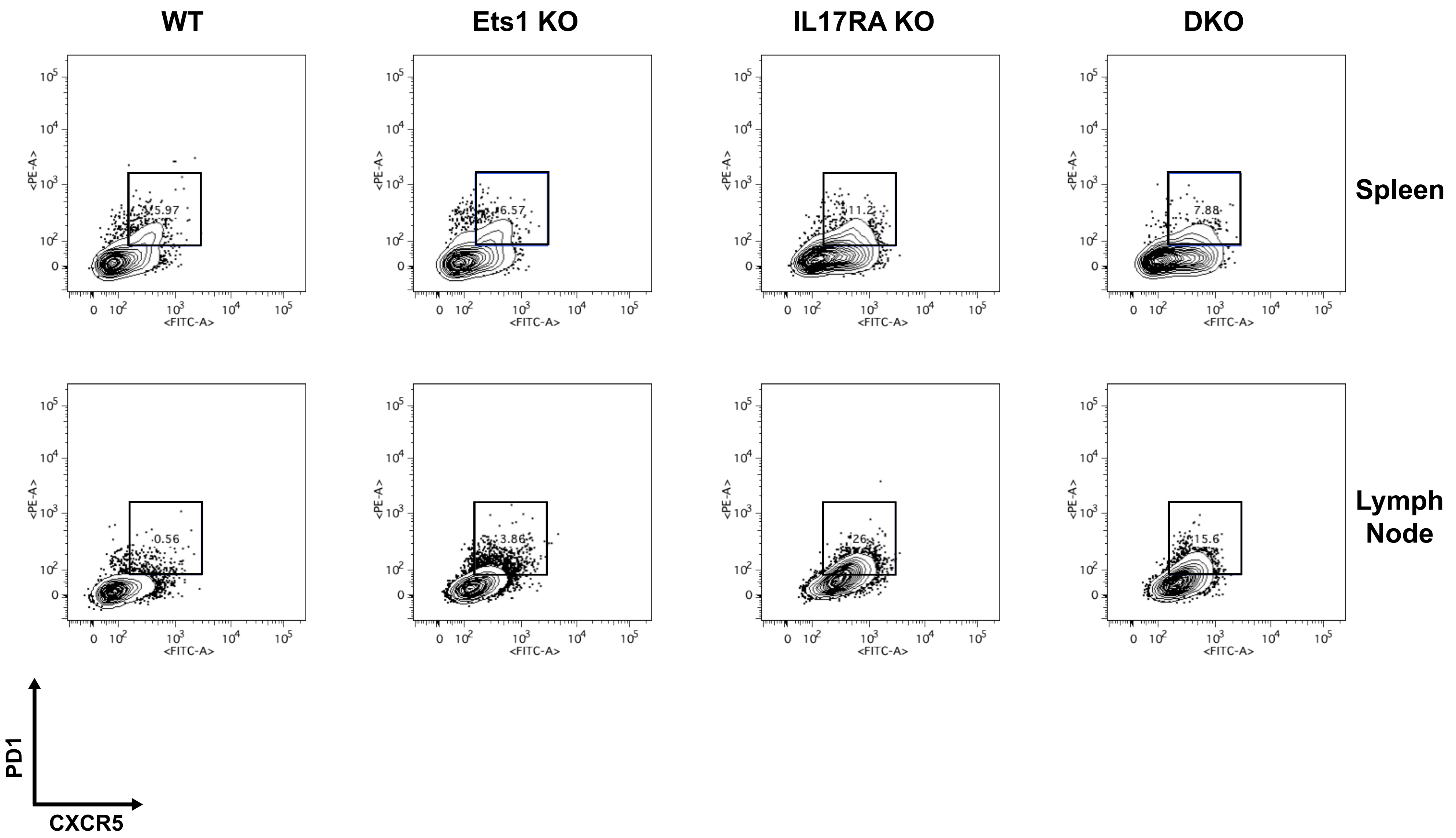

Supplemental Figure 6

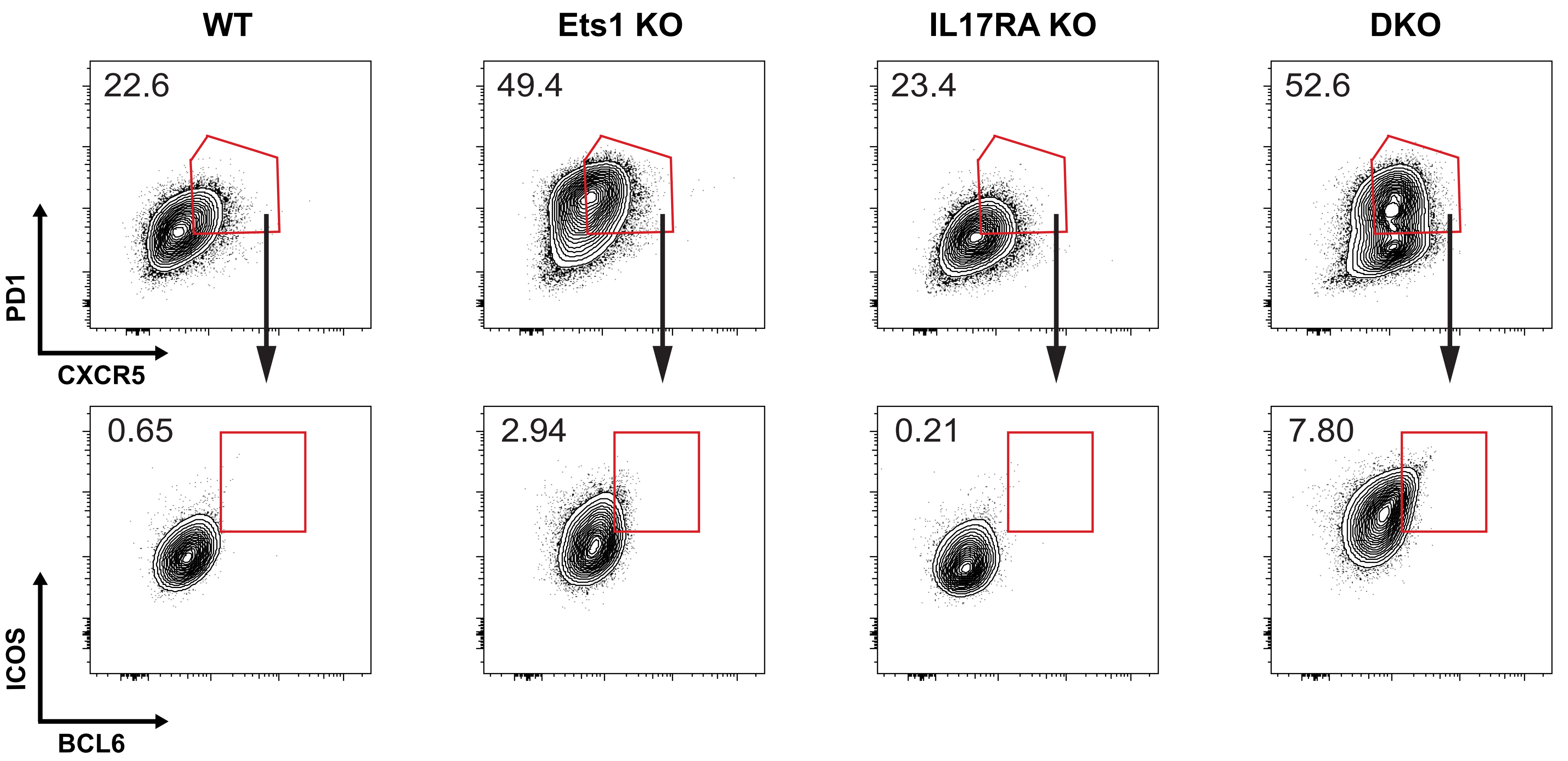

Supplemental Figure 7

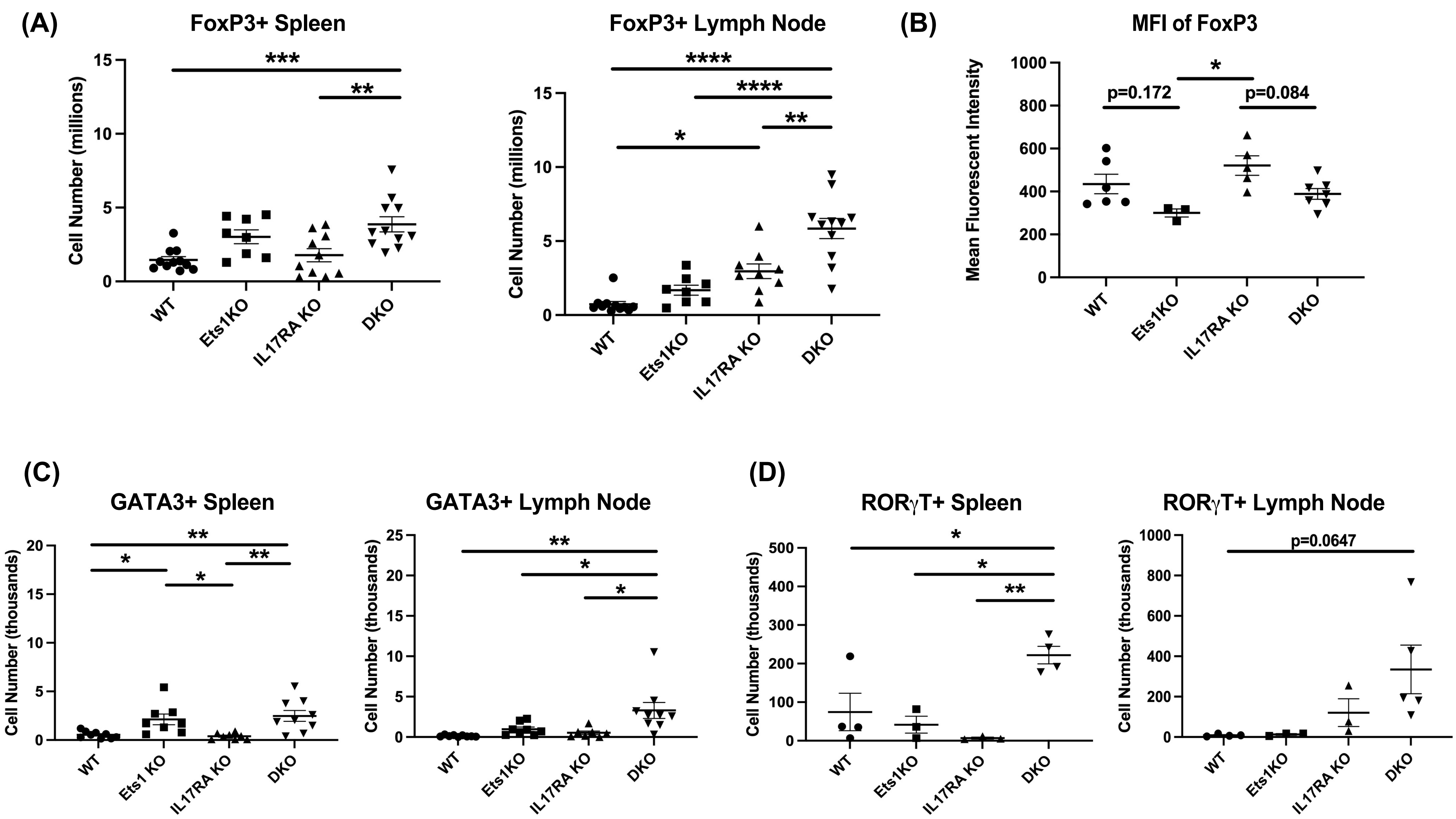

Supplemental Figure 8

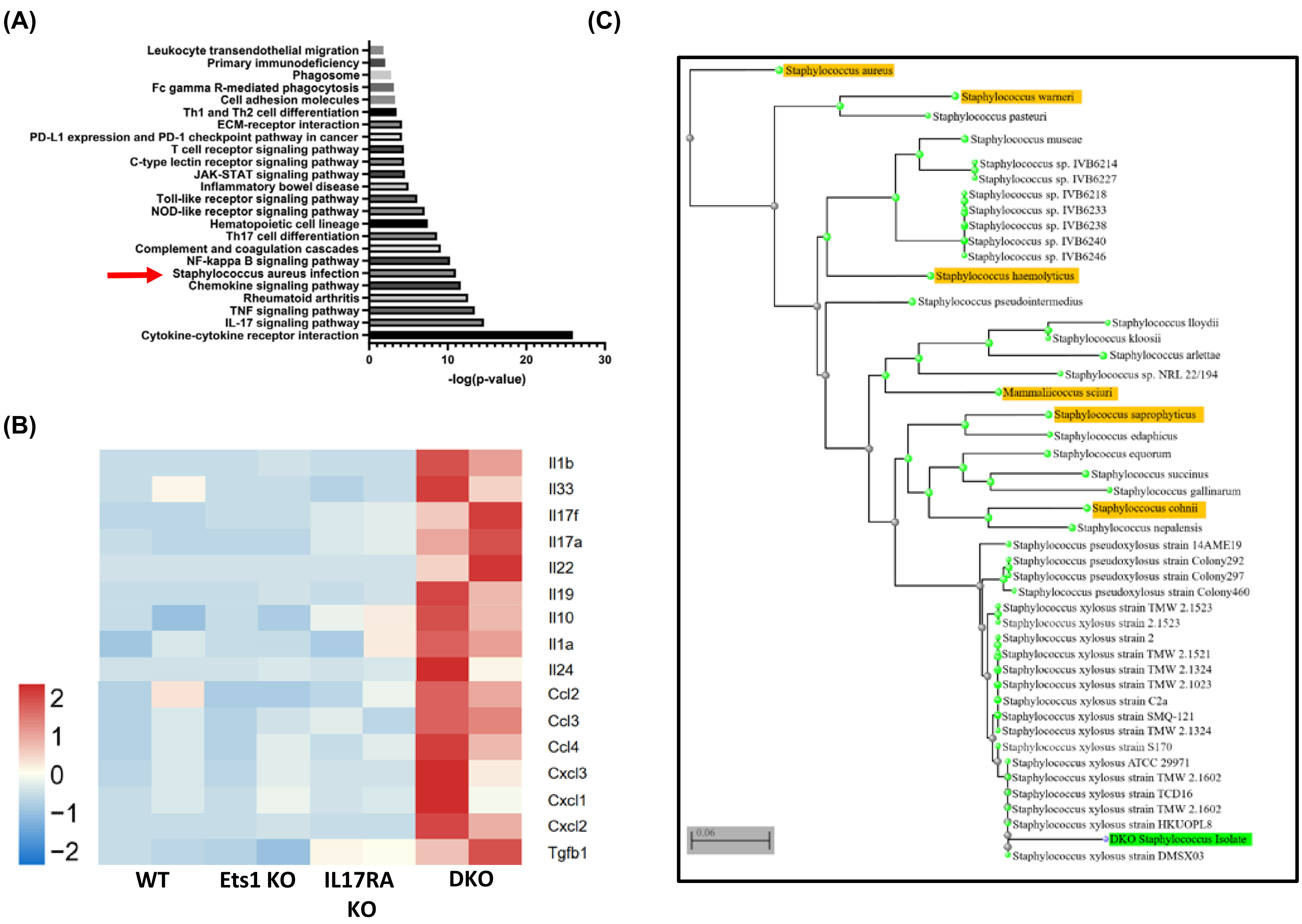

Supplement: Supplementary file 1 [file DataSheet_1.pdf]
